# A Plan for Getting Work

**Published:** 1875-12

**Authors:** 


### A PLAN FOR GETTING WORK.

There is an association of young persons at Springfield, Vt., known as the Industrial Works, that furnishes all its members with constant work at fair wages, and a pleasant home at small cost, which is meeting with success so marked as to attract the attention of all thinking people. The members of this association are all young people who are willing to rid themselves of all bad habits, work steadily, dress economically, and save a portion of their wages; no others are taken. All the men who join are required to furnish a small amount of capital, and save one-fourth of their wages, which must be invested in the capital stock of the association. Women are not required to furnish any capital in the outset, must save one-sixth of their wages and invest it in the business. Those who do not comply with the requirements of the association are expelled, and those who wish to leave can do so at any time, and can withdraw their capital at any time by giving six months' notice. The wages paid to each member is fixed by a board of directors and is proportioned to their skill and ability. They have a large dwelling or home where the members live and enjoy many privileges and comforts not usually found in families or boardinghouses. All pay a moderate price for their board from their wages. They have two new factory buildings, and a good water privilege and considerable machinery, and are engaged in the manufacture of toy and house-furnishing goods for which they find a ready sale. They commenced business a year ago with five hands, and are now working forty-five; their sales for last month were over three thousand dollars, their pay roll for the month was over twelve hundred dollars, and the saving of wages which was added to the capital of the company was more than three hundred dollars. The average amount saved from the wages of each man in a year is one hundred and fifty dollars, and of each woman fifty dollars. Many of the menbers have saved much more than this during the year, but this is all that is required of them. The aggregate amount saved by the present company in a year will be nearly five thousand dollars.

---

The British Empire.—Twenty-five written and various unwritten languages are spoken through the British colonies. There are about 5,000,000 Hindoos, 20,000,000 Mahomedans, 10,000,000 Buddhists, and millions of other idolaters of various descriptions, in the British foreign possessions. The whole population is estimated at 120,000,000. Of these not more than 25,000,000 eat flesh abundantly, about 10,000,000 sparingly; 24,000,000 occasionally and about 70,000,000 live principally on vegetables and fish. About 34,000,000 make wheat, oats, and barley their principal granivorous feed; 19,000,000 potatoes, pulse, and other vegetables, and 10,000,000 rice, maize millet, etc. About 10,000,000 drink wine frequently; 25,000,000 malt liquors; and 60,000,000 chiefly water.

